# Lucy's Flat Feet: The Relationship between the Ankle and Rearfoot Arching in Early Hominins

**DOI:** 10.1371/journal.pone.0014432

**Published:** 2010-12-28

**Authors:** Jeremy M. DeSilva, Zachary J. Throckmorton

**Affiliations:** 1 Department of Anthropology, Boston University, Boston, Massachusetts, United States of America; 2 Department of Anthropology, University of Wisconsin, Madison, Wisconsin, United States of America; University of Delaware, United States of America

## Abstract

**Background:**

In the Plio-Pleistocene, the hominin foot evolved from a grasping appendage to a stiff, propulsive lever. Central to this transition was the development of the longitudinal arch, a structure that helps store elastic energy and stiffen the foot during bipedal locomotion. Direct evidence for arch evolution, however, has been somewhat elusive given the failure of soft-tissue to fossilize. Paleoanthropologists have relied on footprints and bony correlates of arch development, though little consensus has emerged as to when the arch evolved.

**Methodology/Principal Findings:**

Here, we present evidence from radiographs of modern humans (n = 261) that the set of the distal tibia in the sagittal plane, henceforth referred to as the tibial arch angle, is related to rearfoot arching. Non-human primates have a posteriorly directed tibial arch angle, while most humans have an anteriorly directed tibial arch angle. Those humans with a posteriorly directed tibial arch angle (8%) have significantly lower talocalcaneal and talar declination angles, both measures of an asymptomatic flatfoot. Application of these results to the hominin fossil record reveals that a well developed rearfoot arch had evolved in *Australopithecus afarensis*. However, as in humans today, *Australopithecus* populations exhibited individual variation in foot morphology and arch development, and “Lucy” (A.L. 288-1), a 3.18 Myr-old female *Australopithecus*, likely possessed asymptomatic flat feet. Additional distal tibiae from the Plio-Pleistocene show variation in tibial arch angles, including two early *Homo* tibiae that also have slightly posteriorly directed tibial arch angles.

**Conclusions/Significance:**

This study finds that the rearfoot arch was present in the genus *Australopithecus*. However, the female *Australopithecus afarensis* “Lucy” has an ankle morphology consistent with non-pathological flat-footedness. This study suggests that, as in humans today, there was variation in arch development in Plio-Pleistocene hominins.

## Introduction

The longitudinal arch is a unique human structure that helps store elastic energy [1] and maintains the structural rigor of the foot during the push-off stage of bipedal locomotion [2]. Furthermore, the longitudinal arch acts as a shock absorber, mitigating ground reaction forces generated during the foot flat stage of the gait cycle. Though framed by the geometry of the pedal skeleton, the arch itself is ligamentous [3], with contributions from the short and long plantar ligaments, the calcaneonavicular (spring) ligament, bifurcate ligament, and perhaps most importantly, the plantar aponeurosis [4]. The arch is also supported by the intrinsic muscles of the foot and activity of the *m. tibialis posterior*, *m. fibularis longus*, *m. tibialis anterior*, and the deep digital flexors. All primates possess a transverse arch, but only humans have a longitudinal arch making non-human primates anatomically and functionally flat-footed. Despite the importance of the arch to foot biomechanics in modern humans, some people can walk normally and pain free on asymptomatic, physiologic flat feet [5].

Little consensus has emerged regarding the timing, tempo, and pattern of arch evolution in the hominin lineage. This contentious topic has suffered in part from the lack of associated pedal fossils of early hominins. However, researchers have also not agreed on which skeletal anatomies can be reliably correlated with the presence or absence of an arched foot, and even fossil footprints have been interpreted in various ways.

Some have suggested that the 3.0–3.7 Myr-old hominin *Australopithecus afarensis* did not have a longitudinal arch. This assertion is based on the inclination of the facets of the pedal joints [6], and the presence of a robust navicular tuberosity that may reflect weight bearing on the medial side of the foot [7]. However, there is evidence for the calcaneonavicular (spring) ligament in *Au. afarensis* [8], a structure that supports the talar head in an arched foot. The lateral tarsometatarsal joint appears to be rigid [9], suggesting the presence of the long plantar ligament, another important soft-tissue component of the longitudinal arch. Furthermore, 3.6 Myr-old footprints from Laetoli, Tanzania may provide evidence of an arch in *Au. afarensis* [10]–[12]. Others concur that the makers of the Laetoli prints had an arched foot, but hypothesize that they were made by a hominin other than *Au. afarensis* [13]. Still others do not see the makers of the Laetoli trackway as possessing particularly well developed arches [14].

In the 2.0–2.8 Myr-old South African species *Australopithecus africanus*, tarsometatarsal immobility laterally [9], and a weakly developed navicular tuberosity medially [7] both suggest the presence of a stiff, arched foot, as does a slight plantar inclination to the cuboid facet on the StW 352 calcaneus (pers. obs.). The complete set of tarsal and metatarsal bones comprising the OH 8 foot (1.85 Myr-old) has been difficult to interpret with some finding evidence for an arch [6], [9], [15], [16] and others suggesting that the arch was not well developed [17]. Fossil footprints from Ileret, Kenya provide strong evidence for a human-like arched foot by 1.53 Myr-ago [14]. These footprints are consistent with analysis of the pedal remains of *Homo* from Dmanisi suggesting, based on metatarsal torsion, the presence of a well-developed longitudinal arch [18].

**Figure 1:**
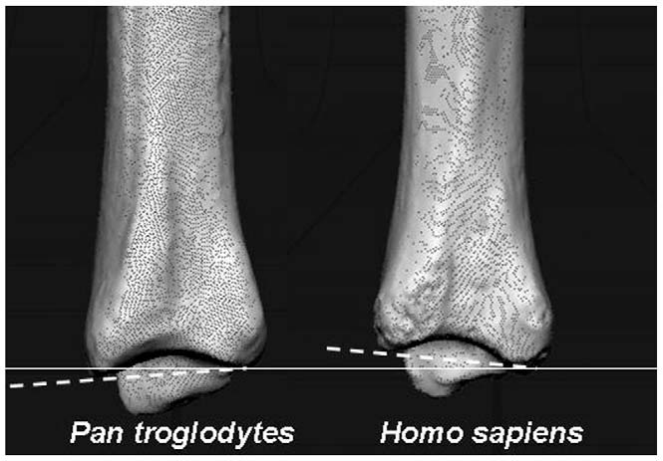
Tibial arch angle in chimpanzee and human. Humans and non-human primates have distinct tilts to the distal tibia in the sagittal plane. In non-human primates (left, chimpanzee), the anterior rim of the tibia (to the left in the figures) is more inferiorly projecting than the posterior rim, creating a posteriorly directed set to the ankle. In humans (right), the posterior rim is more inferiorly projecting, creating an anteriorly directed set to the ankle. In this image, the thin white line has been drawn through the inferomost projection of the posterior rim of both tibiae and is perpendicular to the long axis of the tibia. The tibial arch angle is formed between this white line and the dotted white line intersecting the anterior rim (negative in chimpanzee; positive in humans).

It is in this context that we suggest another potential skeletal correlate of rearfoot arching in the hominin lineage: the tibial arch angle (Figure 1). Viewed laterally, a line connecting the anterior and posterior margins of the distal tibia is rarely perpendicular to the bone's long axis [19]. In most non-human primates, the anterior rim is more inferiorly projecting, producing a posteriorly directed set to the ankle joint. In most humans, it is the posterior rim of the tibia that projects more inferiorly, creating an anteriorly directed set. Though most agree on these characterizations of the distal tibia in the sagittal plane, the functional importance of these morphologies has been difficult to assess. Some have argued that a posteriorly directed set is functionally linked to a bent-hip bent-knee bipedal gait, significant arboreality, and even hindlimb suspension [20]. Others have argued that the tibial arch angle is a variable feature of limited functional significance [21]. Here, we hypothesize that the tibial arch angle is a developmental by-product of unequal forces imposed on the distal tibial physis as a result of rearfoot arching. We first test the hypothesis that the tibial arch angle is correlated with arboreality by measuring this angle in non-human apes representing varied locomotor strategies. We next test the relationship between the tibial arch angle and rearfoot arching by measuring these variables in radiographs of modern human feet (n = 261). These results are then applied to the fossil record to reevaluate hypotheses regarding arch evolution in the hominin lineage.

## Materials and Methods

The tibial arch angle was taken on lateral view photographs (Nikon D100 digital camera) of tibiae from adult, wild-shot primates listed in Table 1. This angle was also taken on human skeletal material from the Libben population (Kent State University), Hamann-Todd (Cleveland Museum of Natural History), and an unprovenienced population from the Department of Anthropology at the University of Michigan. The images were imported into Image J, and the tibial arch angle was measured by taking the angle formed between a line drawn from the inferomost projection of the posterior tibial rim to the inferomost projection of the anterior tibial rim, and a line perpendicular to the long axis of the tibial shaft. This angle was measured to the nearest whole degree. This same measurement was taken on photographs taken in lateral view of casts and original fossil material listed in Table 2.

**Table 1:** Extant tibiae measured in this study.

Species	Male	Female	Sex unknown	Total
*Homo sapiens* (radiographs)	-	-	-	261
*Pan troglodytes*	18	20	10	48
*Gorilla gorilla gorilla*	23	19	3	45
*Gorilla gorilla beringei*	15	6	1	22
*Pongo pygmaeus*	12	19	8	39

**Table 2:** Fossil tibiae measured in this study.

*Specimen	Age	Species designation	#Tibial arch angle (°)	References
KNM-KP 29285	4.12	*Australopithecus anamensis*	−1.8	[22]
A.L. 333-6	3.2	*A. afarensis*	2.9	[23], [24]
A.L. 333-7	3.2	*A. afarensis*	5.5	[23], [24]
A.L. 288-1	3.18	*A. afarensis*	−5.0	[23], [25]
StW 358	2.0–2.6	*A. africanus*	4.2	[26], [27]
StW 389	2.0–2.6	*A. africanus*	3.7	[26], [27]
KNM-ER 1481	1.9	*H. habilis? H. erectus?*	−2.1	[28]–[30]
KNM-ER 1500	1.9	*A. boisei?*	3.7	[28], [31]
KNM-ER 2596	1.9	Hominin	0.8	[28], [32]
†OH 35	1.85	*H. habilis?*	4.8	[33]
StW 567	1.4–1.7	*Homo*	−3.0	[34], [35]
KNM-WT 15000	1.5	*H. erectus*	1.8	[36]

*All original fossils except for A.L. 333-6, A.L. 333-7, and A.L. 288-1.

# Positive tibial arch angles indicate an anteriorly directed set; negative tibial arch angle indicate a posteriorly directed set.

† There is damage to the anterior rim of OH 35, and this value should thus be regarded as an estimate. The tibial arch angle reported here assumes that the anterolateral edge of the tibial rim accurately reflects the most inferior projection of this surface.

Over 300 lateral weight-bearing radiographs (taken as part of routine medical care) of a modern, habitually shod population were surveyed for relevance to the study. These x-rays were completely deidentified prior to analysis, in full compliance with HIPAA laws. Radiographs of skeletally immature individuals, as well as those suffering from advanced diabetic neuropathy, Charcot-Marie-Tooth neuropathy, and other conditions that compromise normal foot biomechanics were excluded. Further, radiographs exhibiting insufficient resolution for the rapid and unambiguous identification of relevant osteological landmarks (e.g. medial malleolus obscuring the outline of the distal tibia set) were also excluded. All measurements were taken of the right foot using standard equipment (i.e. viewing box, straightedge, compass).

**Figure 2:**
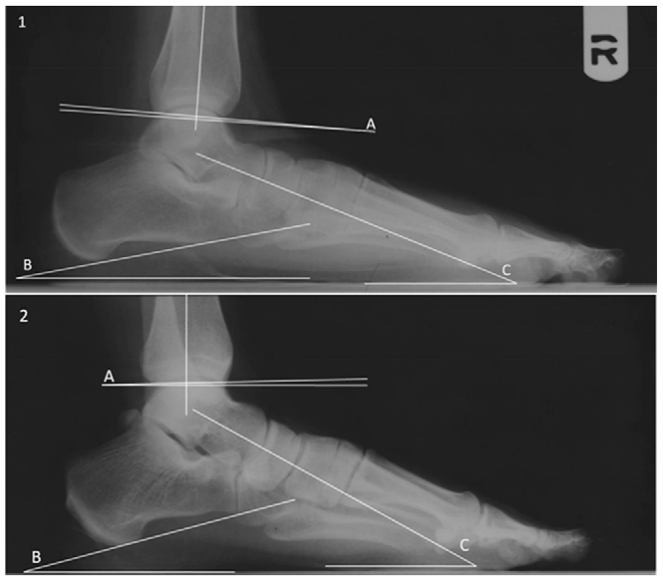
Measurements taken in this study. 1. A flat foot exhibiting a posteriorly directed tibial set. 2. An arched foot exhibiting an anteriorly directed tibial set. Both 1 and 2: A. Tibial arch angle. B. Calcaneal inclination angle. C. Talar declination angle.

The three measurements collected were the calcaneal inclination (CI), talar declination (TD), and distal tibial arch angle (TAA). CI was determined by drawing a line connecting the plantarmost point of the anterior face of the calcaneus (the calcaneo-cuboid articular facet) to the plantarmost point of the calcaneal body relative to the substrate (Figure 2). TD was determined by drawing a line bisecting the most dorsal and plantar points of the talar head and the bisection of the narrowest point of the talar neck, also relative to the substrate. The talocalcaneal angle was calculated as the sum of CI+TD. TAA was determined by drawing a line from the inferomost projection of the posterior tibia to the inferomost projection of the anterior tibia relative to the line drawn perpendicular to the axis of the tibial shaft. The relationship between the tibial arch angle and the talar declination, calcaneal inclination and talocalcaneal angles was elucidated using a non-parametric Mann-Whitney U-test in SPSS 16.0. We also tested whether the TAA and the TD and CI covaried using a Pearson correlation test (SPSS 16.0). Comparisons of the tibial set in extant primates were evaluated with a Student's t-test in Microsoft Excel.

Resampling statistics were employed to test the likelihood of sampling the tibial arch angles present in the fossil tibial sample (n = 12) from a modern human population. In the fossil tibial sample, five specimens (KNM-KP 29285, AL 288-1, KNM-ER 2596, KNM-ER 1481, and StW 567) have a posteriorly directed or neutral tibial arch angle. Twelve measured tibial arch angles were randomly selected with replacement from the modern human radiographic sample. Out of these twelve, the number of individuals with a posteriorly directed, or neutral tibial arch angle were identified and summed. A neutral tibial arch angle was defined as an angular measurement between −1° and 1°. This process was repeated 5000 times to test the likelihood of having five neutral or posteriorly directed tibial arch angles if only 12 tibiae are randomly sampled.

## Results

**Figure 3:**
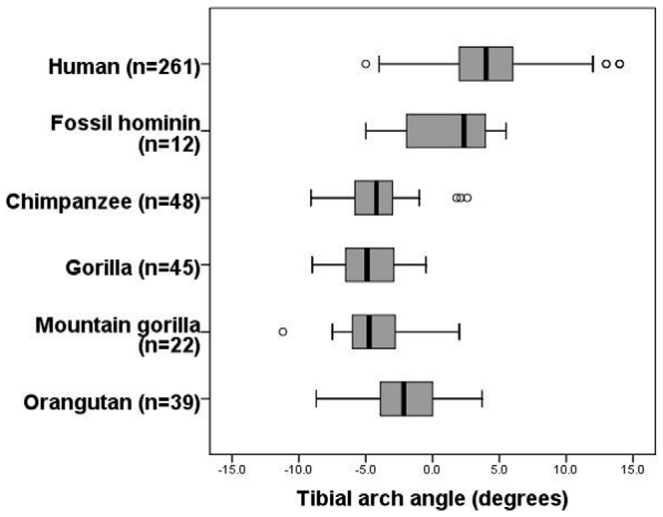
Variation in tibial arch angle in extant apes and fossil hominins. The tibial arch angle differentiates humans and non-human primates. Mountain gorillas, lowland gorillas, and chimpanzees have statistically indistinguishable tibial arch angles, and orangutans have the least posteriorly directed angle of the great apes. These comparative data do not support the hypothesis that this angle is related to arboreality or hindlimb suspensory abilities. Instead, it is argued in this study that the tibial arch angle is related to rearfoot arching. Humans are quite variable for this measure, and fossil hominins occupy the lower end of the modern human spectrum, though this distribution can be sampled from a modern human population. The median (black bar), interquartile range (box) and overall ranges (whiskers) are illustrated. Outliers defined as 1.5 times the interquartile range are shown as circles.

The tibial arch angle is statistically identical between the highly terrestrial mountain gorilla (*Gorilla gorilla beringei*) and the more arboreal lowland gorilla (*Gorilla gorilla gorilla*) (t = 0.65; p = 0.52) (Figure 3). Furthermore, both gorilla species have a statistically identical tibial arch angle with the more arboreal chimpanzee (*Pan troglodytes*) (t = 1.24; p = 0.22). The most arboreal of the great apes, the orangutan (*Pongo pygmaeus*) has a statistically distinct tibial arch angle from the African apes (t = 4.80; p<0.001). However, it is in the opposite direction as expected, producing a less posteriorly directed set than that found in African apes (Figure 3). These results are consistent with others [19], and suggest that the tibial arch angle has little to do with arboreality or hindlimb suspension. Humans have the most anteriorly directed tibial arch angle, statistically distinct from all ape taxa measured in this study (p<0.001 for all comparisons).

**Figure 4:**
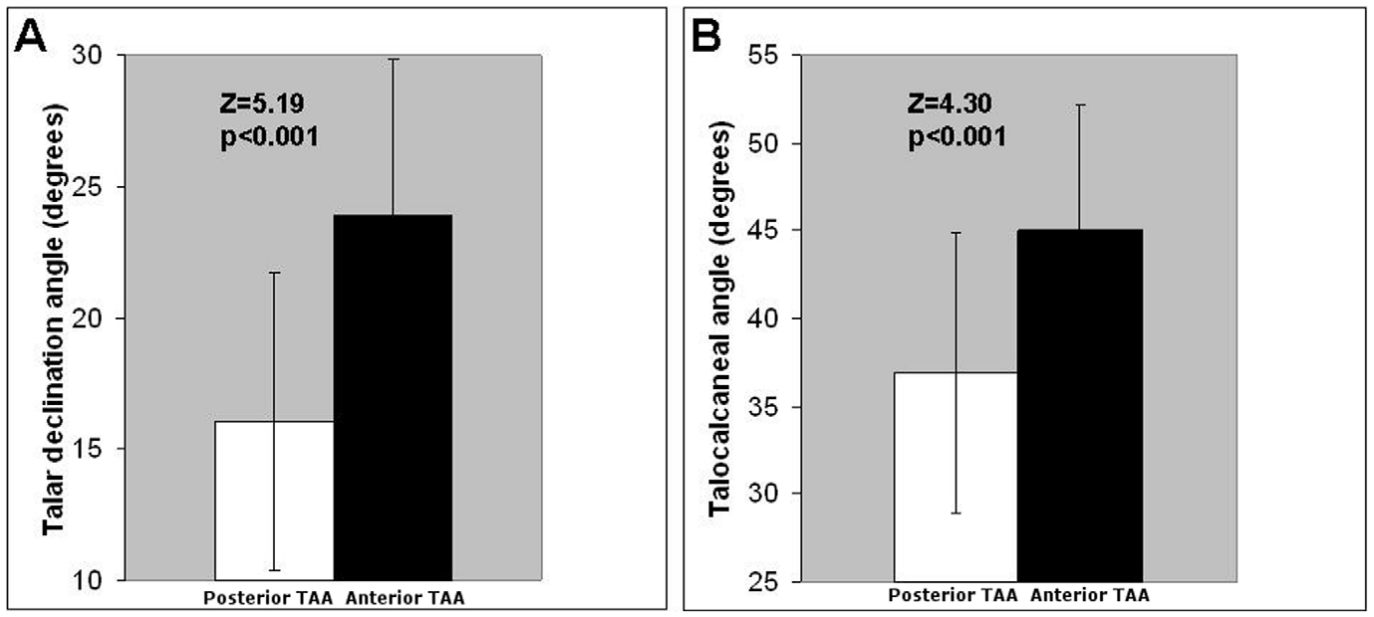
Relationship between tibial arch angle and rearfoot arching in humans. Modern humans with a Lucy-like posteriorly directed set to the distal tibia (white bars mean ± sd) have significantly lower talar declination (A) and talocalcaneal angles (B) than modern humans with an anteriorly directed set to the ankle joint (black bars mean ± sd).

**Figure 5:**
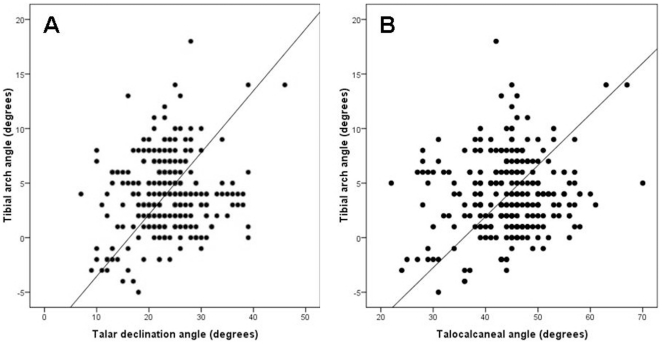
Correlation between tibial arch angle and measures of flat foot in humans. There is a statistically significant positive correlation between the tibial arch angle and two measures of asymptomatic flat-footedness, the talar declination angle (A), and the talocalcaneal angle (B). A regression line generated using reduced major axis regression is drawn in each graph.

To test the hypothesis that the tibial arch angle is instead related to rearfoot arching, 261 weight-bearing radiographs of human ankles taken in lateral view were examined. A posteriorly directed set to the ankle is present in 8% of the radiographic sample, including one individual with a set as great as that found in Lucy (5°). An “even” tibial set (0°) is present in 5% of the population, while most (86%) have an anteriorly directed set to the ankle. Those with a posteriorly directed tibial arch angle have significantly lower talocalcaneal (z = 4.3; p<0.001) and talar declination angles (z = 5.19; p<0.001) (Figure 4) than those with an anteriorly directed set. There was no statistically significant difference in the calcaneal inclination angle between those with a posteriorly directed set and those with an anteriorly directed set. Using parametric statistics, it was found that the tibial arch angle and the talar declination angle are correlated (r = 0.21, p = 0.001), as are the tibial arch angle and the talocalcaneal angle (r = 0.16, p = 0.009) (Figure 5). The tibial arch angle was not correlated with the calcaneal inclination angle (r = 0.03, p = 0.61).

**Figure 6:**
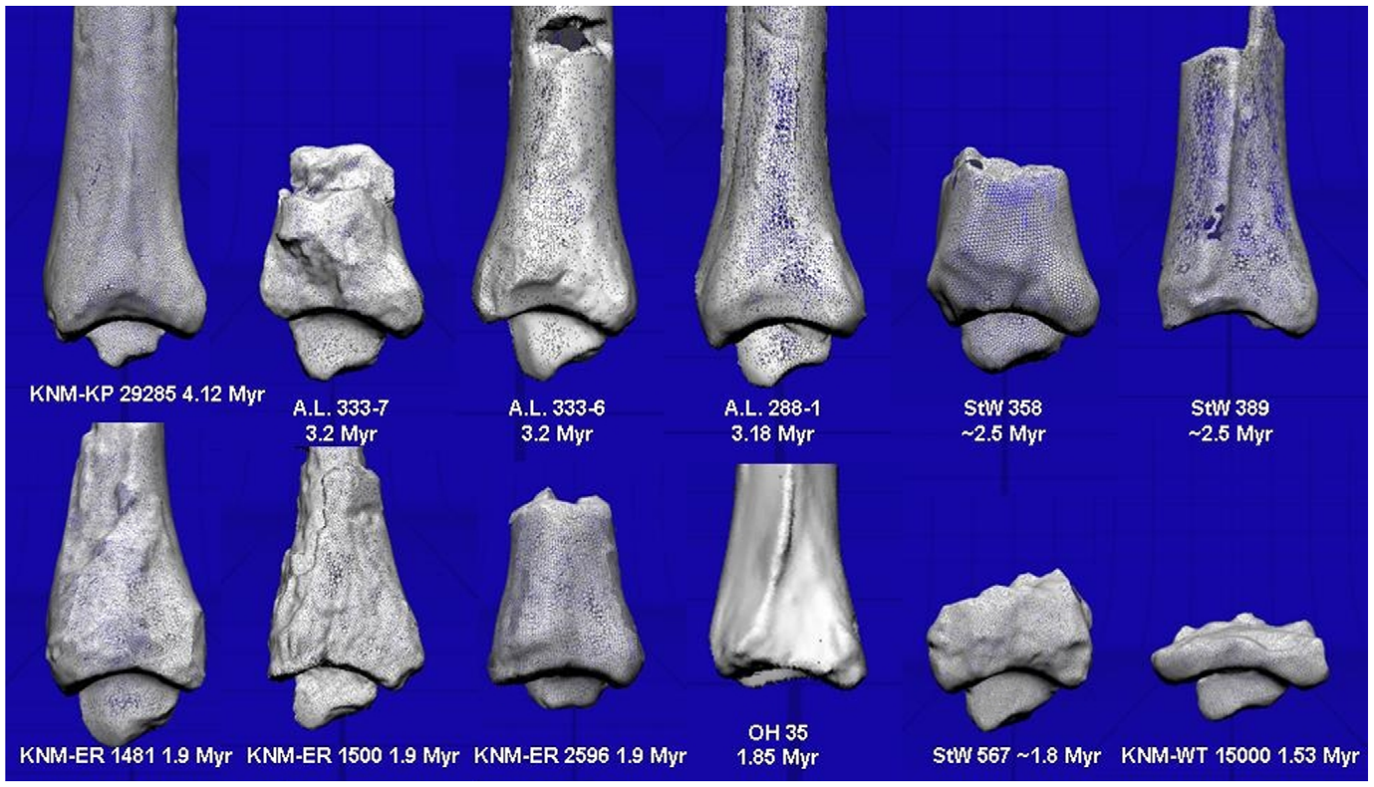
Fossil hominin distal tibiae. Fossil hominin tibiae examined in this study with genus *Australopithecus* in the top row, and *Homo* and *Paranthropus* in the bottom row. All are scans of original fossils with the exception of the three fossils from Hadar, Ethiopia (A.L. fossils), and OH 35. Fossils were 3D laser scanned, scaled to roughly the same size, and presented here to visualize the tibial arch angle. Anterior is to the left, posterior to the right. KNM-KP 29285, A.L. 288-1, and StW 567 have been reversed to reflect the left side. Individual arch angles are presented in Table 2. Notice here the posteriorly directed set to A.L. 288-1, and the slight posteriorly directed set to KNM-KP 29285, StW 567, and KNM-ER 1481. All other fossils show an anteriorly directed set.

There is great variation in the tibial arch angle in fossil hominins (Table 2). Some have a near “even” tibial set, while most possess a human-like anteriorly directed set, including two fossils from *Au.afarensis*. Four distal tibiae possess a posteriorly directed tibial arch angle. Most prominent among these is the female *Au. afarensis* A.L. 288-1, or, “Lucy”. The other tibiae with a slightly posteriorly directed tibial arch angle are the 4.12 Myr-old tibia from *Au. anamensis*, KNM-KP 29285, and Pleistocene fossils attributed to the genus *Homo*: KNM-ER 1481 and StW 567 (Figure 6). KNM-ER 2596 has a neutral tibial arch angle.

Using resampling statistics, the likelihood of having five posteriorly directed or neutral tibial arch angles represented in a randomly selected group of twelve tibiae is 13.8%, well above the 5% required for statistical significance.

## Discussion

The results of this study suggest that a critical adaptation for bipedality in modern humans, the longitudinal arch, may have been variably present in *Australopithecus* by 3.2 Myr-ago, and perhaps earlier. However, as in humans today, Lucy's posteriorly directed set to the ankle may indicate a low rearfoot arch. The relationship between the tibial arch angle and the talar declination and the talocalcaneal angles are statistically significant using both parametric and non-parametric statistics, though the calcaneal inclination angle alone is not correlated with the tibial arch angle.

Hypotheses suggesting that the posteriorly directed tibial arch angle is related to arboreality and hindlimb suspension are not supported by comparative data (Figure 3) given that there is no statistical difference between the mountain gorilla and the lowland gorilla despite differences in arboreality. Furthermore, the most arboreal of the apes, the orangutan, has a more human-like tibial arch angle than the more terrestrial African apes. This result is surprising, and the reason for a less posteriorly directed tibial arch angle in orangutans is unclear, though perhaps related to differences in foot and ankle loading patterns during a more diverse repertoire of clambering, climbing, bridging and suspensory arboreal activities in orangutans [37], relative to African apes. What does not explain these data is allometry. In both humans and non-human primates, there is no relationship between the tibial arch angle and the width of the tibial plafond, a proxy for size (Figure S1). Thus, the posteriorly directed tibial arch angle in “Lucy” is not simply a by-product of her small size, but more likely to be a result of her asymptomatic flat footedness.

Though we find with these data that “Lucy” may have had a flat foot, this does not imply she suffered from pathological flat foot (*pes planus*), in which the arch collapses. *Pes planus* is often typified by a high, rather than a low, talar declination angle, in part because the spring ligament no longer supports the talar head [38], and thus these findings are in concert with other reports that pathological and asymptomatic *pes planus* are radiologically distinguishable [39]. It is important to note as well that Lucy may have suffered from a spinal pathology, best characterized as Scheuermann disease [40]. How this may have impacted her arch development, tibial arch angle, and gait is currently unstudied. An important finding in this study is that asymptomatic flat-footedness did not characterize the species *Au. afarensis*, and instead may just describe the foot of one specific female, Lucy. Two other distal tibiae from Hadar, Ethiopia, A.L. 333-6 and A.L. 333-7 (Figure 6), have distinctly human-like anteriorly directed sets to the distal tibia, implying the presence of rearfoot arching. These two individuals are more like the makers of the 3.6 Myr-old Laetoli footprints, argued to have been made by hominins possessing a well-developed longitudinal arch [11] but see [14]. An anteriorly directed set to the ankle is also present in the two *Au.africanus* fossils measured in this study (Table 2; Figure 6), suggesting a rearfoot arch in this South African hominin as well. However, variation likely exists in the S. African hominins given what appears to be a posteriorly directed set in a published photograph of the tibia from “Little Foot” StW 573 [41].

Interestingly, a slight but measureable posteriorly directed set to the ankle is present in a higher percentage of fossil hominin tibiae (33%; n = 4/12) than in modern humans. This posteriorly directed set includes two tibiae thought to be from the genus *Homo*: StW 567 [34], [35], [42], and KNM-ER 1481 [29], [30]. These results appear to suggest that asymptomatic flatfootedness may have been more frequent in Plio-Pleistocene hominins than in modern humans. However, the use of resampling statistics demonstrates that this is more likely a sampling issue, as this frequency of posteriorly directed and neutral tibial arch angles can be sampled from a modern human population 13.8% of the time.

In our sample of modern human radiographs, we find that 8% possess a posteriorly directed set to the ankle. This is a considerably higher percentage than previous studies which found a posteriorly directed set in only 1.5% of a large skeletal sample [20]. Examination of additional skeletal collections suggests that the development of the tibial arch angle may vary by population (Table 3). There was only one individual (2.2%; n = 1/45) with a posteriorly directed angle of only −1.0° in the Libben population. However, a higher percentage was found in an unprovenienced population from the University of Michigan Department of Anthropology (7.6%; n = 5/66), and the Hamann-Todd collection (12.5%; n = 3/24), including some with posteriorly directed angles as great as −4.0°. These findings of population-level differences are consistent with evidence of variation in foot arch development from one population to another [43]. If the 8% found in this study does prove to be higher than the typical human population, the hypothesis that flatfootedness was more common in early hominins should be revisited. It remains possible that an increase in the frequency of arched feet may have evolved with later genus *Homo* (e.g. *H. erectus*) [44], perhaps owing to the benefits of returning elastic energy to the foot while running [45]. For now, however, the data presented in this study suggest that Plio-Pleistocene hominins had the same variation in tibial arch angle as that found in modern humans.

**Table 3:** Population level differences in tibial arch angle (TAA).

Population	Sample size	Tibial arch angle (mean ± sd)	% of sample with posteriorly directed TAA	Minimum tibial arch angle	Maximum tibial arch angle
Radiographs	261	4.0°±3.6°	8.4%	−5.0°	18.0°
Hamann-Todd	24	3.0°±2.7°	12.5%	−3.0°	8.0°
Unprovenienced- University of Michigan	66	2.8°±2.3°	7.6%	−4.0°	8.0°
Libben	45	5.9°±3.1°	2.2%	−1.0°	13.0°

We hypothesize that the mechanism linking the tibial arch angle with rearfoot arching follows Hueter–Volkmann's “law” in which compressive forces can inhibit chondral growth [46]. At birth, humans do not have a structural arch. The arch becomes noticeable in most children by the ages of 3–6, though it does not develop in all children [47]. As the arch develops, the anterior tibial physis may receive more force than the posterior region. This arrangement may promote an uneven amount of chondral proliferation posteriorly given the reduction or cessation of chondral growth in regions of elevated pressure anteriorly. This differential growth is critical for maintaining the plane of the ankle joint perpendicular to ground reaction forces [48]. We therefore hypothesize that this morphology is not strictly determined by developmental growth fields and instead is reflective of forces imposed on the foot during the juvenile period. The tibial arch angle may therefore be one of a suite of characteristics suggested to be skeletal correlates of arch formation in hominins.

How the presence or absence of an arch impacts the locomotor performance of the individual in question is important to consider. Many humans today have asymptomatic physiologic flat feet, and suffer no ill effects [5]. Despite the prevailing view that flatfeet are a cause for concern [5], many recent studies have shown that individuals with low arches are no more likely to suffer injury or pain than individuals with high arches [49]–[51]. Locomotor performace also appears to be indistinguishable between teenagers with flat feet and those with normal arches [52]. However, arch development and the form of an individual's foot do affect lower limb kinematics [53], and these changes can increase the risk of injury during rigorous physical activity, such as distance running [54]. Additionally, arch structure has been shown to impact patterns of plantar pressure [55], [56]. For example, the arch helps to shift forces laterally in the human foot [48], and thus the relatively enlarged lateral metatarsal heads, present in *Au. afarensis* [57], may provide additional skeletal evidence for an arch. Nevertheless, additional fossil material from the pedal skeleton, and continued study of recovered pedal remains, will both be necessary to reconstruct foot morphology, arch development, and locomotor biomechanics in early hominins.

### Conclusion

As in humans today, *Australopithecus* exhibited variation in foot morphology and arch development. Despite having only preserved the talus and two phalanges, we suggest that it is the distal tibia that provides evidence for foot structure in the “Lucy” skeleton. Our findings suggest that this female *Au. afarensis* possessed an asymptomatic physiologic flatfoot, though two other tibiae from Hadar, Ethiopia suggest the presence of a rearfoot arch in this species. Whether flat-footedness was more common in early hominins will require additional fossil material, and identification of additional skeletal correlates of the longitudinal arch.

## Supporting Information

Figure S1 Relationship between tibial arch angle and tibial plafond width in humans and apes. The tibial arch angle shows no allometric relationship with the width of the tibial plafond (taken at the midpoint of the talar articular surface). This finding demonstrates that the tibial arch angle in “Lucy” is not a function of her small size, but rather is a product of some other aspect of her foot functional anatomy. We suggest in this paper that it is a skeletal correlate of an asymptomatic flat-foot.
(3.86 MB TIF)
