# Supplementary figures and images for: Lucy's Flat Feet: The Relationship between the Ankle and Rearfoot Arching in Early Hominins
Source: PLoS One. 2010 Dec 28;5(12):e14432. doi: 10.1371/journal.pone.0014432 (PMC3010983; doi:10.1371/journal.pone.0014432)

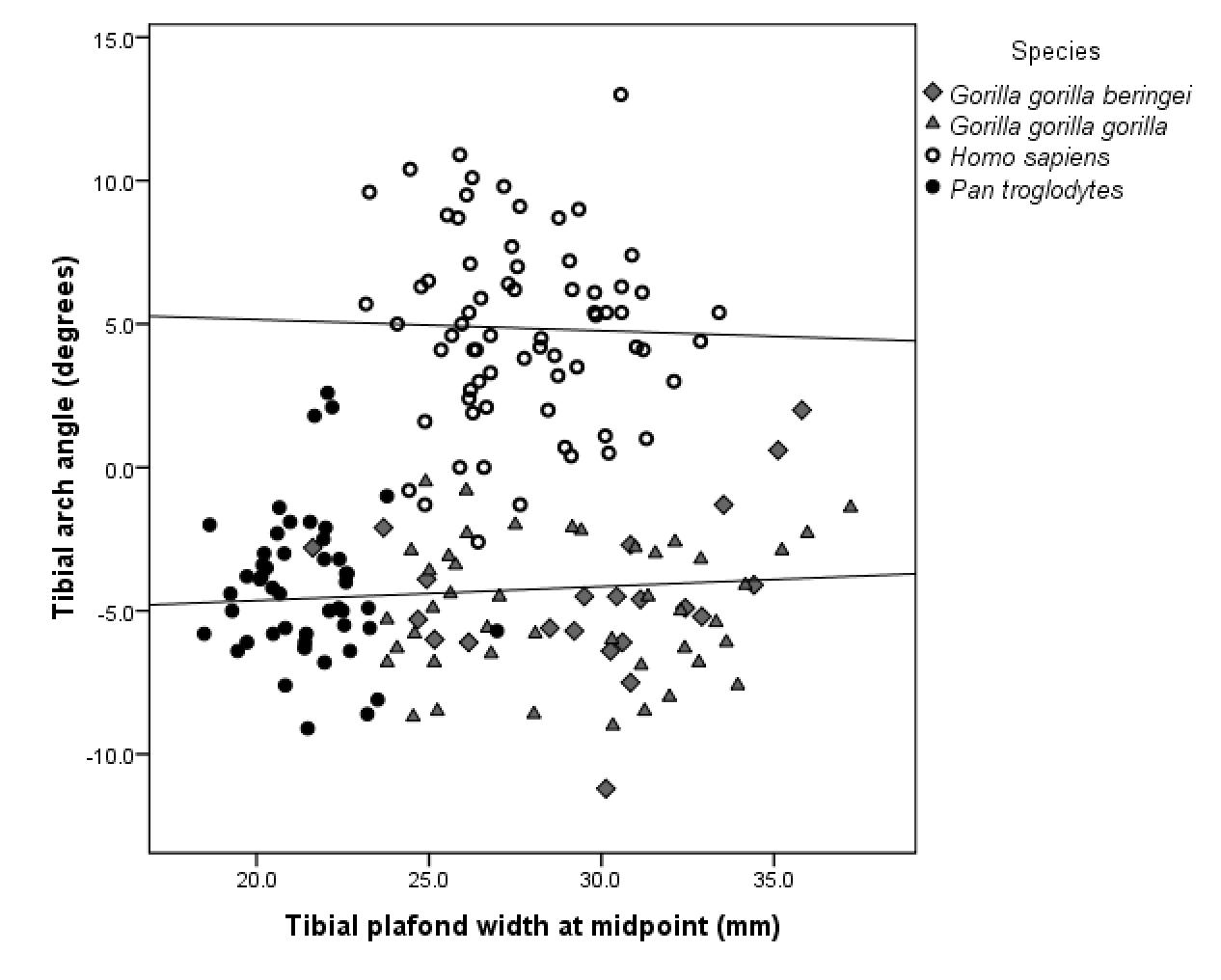

Supplement: Figure S1 — Relationship between tibial arch angle and tibial plafond width in humans and apes. The tibial arch angle shows no allometric relationship with the width of the tibial plafond (taken at the midpoint of the talar articular surface). This finding demonstrates that the tibial arch angle in “Lucy” is not a function of her small size, but rather is a product of some other aspect of her foot functional anatomy. We suggest in this paper that it is a skeletal correlate of an asymptomatic flat-foot. (3.86 MB TIF) [file pone.0014432.s001.tif]
